# Genetics of Hypertension and Cardiovascular Disease

**DOI:** 10.4061/2010/951254

**Published:** 2011-01-24

**Authors:** Tomohiro Katsuya, Stephen B. Harrap, Toshio Ogihara

**Affiliations:** ^1^Department of Clinical Gene Therapy, Osaka University Graduate School of Medicine, Suita 660-0052, Japan; ^2^Osaka General Medical Center, Osaka Prefectural Hospital Organization, Osaka 558-8558, Japan

With the force something like a tsunami wave, a surge of genome-wide association studies (GWASs) in common complex diseases has flooded the recent literature and left us awash with data regarding the identification of causative genetic risk factors for cardiovascular disease such as hypertension. Nevertheless, the more slowly moving tide of the classical candidate gene approach also makes steady progress to clarify the interaction between gene function and pathogenesis of hypertension. Like most other common conditions the estimated effects of each genetic variant on blood pressure and the predisposition to hypertension is quite small, yet the delineation of genetic contribution could reveal new targets for risk reduction in cardiovascular disease.

The main focus of this special issue is to highlight the genetic basis of hypertension and cardiovascular disease and outline recent progress in genomics. In addition, several successful investigations in common diseases are introduced as good examples of the potential for better understanding of hypertension using genomic approaches.

In the first part of this issue, two papers provide clear reviews of the recent progress in the GWAS approach for hypertension and coronary artery disease. These papers elucidate not only the common findings in studies that, to a certain extent, disparate but also interprete these findings in the context of the advantages and limitations of GWAS. This context includes the contrast between the very strong (genome-wide) significance of associations and the relatively small phenotypic effects of each SNP. In terms of discovery potential, unexpected candidate genes obtained from GWAS provide fresh perspectives in hypertension research.

But the genomic picture is for more complex than isolated SNPs. It involves the ways in which the sophisticated organization of the operational elements of the genome interdigitates to provide tissue and development stage-specific regulation and integration of gene expression. It also aligns these factors with phenotypic changes that can result. Two subsequent review articles illustrate these principles by showcasing a new cutting edge of cardiovascular research in the form of microRNAs (miRNAs) and scavenger receptors. Both papers disclose the potentials of these newcomers as therapeutic targets of cardiovascular disease.

In a more focused review of candidate genes, the next 2 papers focus on gene polymorphisms of adrenergic receptors. K. Masuo examines the critical role of beta2- and beta3-adrenoceptors gene polymorphisms on hypertension or metabolic syndrome unequivocally. K. B. Boström et al. report that a polymorphism (Arg389Gly) of beta1-adrenoceptor gene is significantly associated with increased risk of obstructive sleep apnea (OSA) with hypertension.

In the research using candidate gene approach, precise and appropriate phenotyping is important to identify small but significant genetic effect. G. D. Kitsios and E. Zintzaras reveal the protective effect of a haplotype of endothelial nitric oxide synthase gene (NOS3) for predisposition to hypertension. K. Sugimoto et al. point out that a polymorphism in the promoter region of regulator of G protein signaling-2 (RGS2) gene affects the difference of antihypertensive medication in a clinical trial. 

The last two papers extend the consideration of novel and unexpected concepts for cardiovascular research. A recent topic is the awareness of shared physiological mechanisms between osteoporosis and cardiovascular disease. F. Marini and M. L. Brandi elegantly review the genetic aspects of osteoporosis and touch on the shared mechanisms. The last manuscript by G. Mertens offers a unique perspective on these issues by zooming out to take an evolutionary view. To consider the underlying mechanisms of atherosclerosis, we have to take a larger view without becoming lost in details of the immediate.

We hope that this special issue is useful for your research and is conducive to the incorporation of new concepts and methods in your own laboratory.

*Tomohiro Katsuya* *Tomohiro Katsuya* *Stephen B. Harrap* *Stephen B. Harrap* *Toshio Ogihara* *Toshio Ogihara*

